# Practice Change Needed for the Identification of Pediatric Hypertension in Marginalized Populations: An Example From South Africa

**DOI:** 10.3389/fped.2022.877431

**Published:** 2022-05-11

**Authors:** Patricia Arnaiz, Ivan Müller, Harald Seelig, Markus Gerber, Jacob Bosma, Danielle Dolley, Larissa Adams, Jan Degen, Stefanie Gall, Nandi Joubert, Madeleine Nienaber, Siphesihle Nqweniso, Ann Aerts, Peter Steinmann, Rosa du Randt, Cheryl Walter, Jürg Utzinger, Uwe Pühse

**Affiliations:** ^1^Department of Sport, Exercise and Health, University of Basel, Basel, Switzerland; ^2^Department of Human Movement Science, Nelson Mandela University, Gqeberha, South Africa; ^3^Swiss Tropical and Public Health Institute, Allschwil, Switzerland; ^4^University of Basel, Basel, Switzerland; ^5^Novartis Foundation, Basel, Switzerland

**Keywords:** pediatric hypertension, prevalence, identification, normative blood pressure tables, international guidelines, marginalized settings, Africa

## Abstract

**Introduction:**

Hypertension in children has increased globally over the past 20 years; yet, little is known about this issue among disadvantaged communities from low- and middle-income countries. Age-, sex-, and height-adjusted normative tables are the “gold” standard for the diagnosis and estimation of pediatric hypertension worldwide, but it is unclear whether the use of international standards is appropriate for all contexts. The purpose of this study was to evaluate and compare different international references to identify hypertension among South African school-aged children from disadvantaged communities.

**Methods:**

Blood pressure, weight, and height were measured in a cohort of 897 children aged 8–16 years from eight peri-urban schools in the Eastern Cape of South Africa. Cross-sectional prevalence of hypertension was calculated according to American, German, and global normative tables, as well as pseudo-normative data from the own study population. Isolated systolic hypertension and body mass index (BMI) were considered markers for cardiovascular disease. Multinomial logistic regression was used to compare the likelihood of blood pressure categorization with increasing BMI levels.

**Results:**

Hypertension prevalence ranged from 11.4% with the pseudo-normative study tables to 28.8% based on the German reference. Global guidelines showed the highest agreement both among international standards (92.5% with American guidelines) and with the study reference (72.5%). While the global and the American references presented higher systolic over diastolic hypertension rates (23.6 vs. 10.6% and 24.2 vs. 14.7%, respectively), the American guidelines predicted the highest increased risk for hypertension stage 2 [odds ratio, 1.72 (95% confidence interval: 1.43–2.07)] with raising levels of BMI.

**Conclusion:**

Our results support the heterogeneity of blood pressure estimates found in the South African literature, and highlight the underrepresentation of African children in international guidelines. We call for caution in the use of international standards in different contexts and advocate for the development of normative tables that are representative of the South African pediatric population necessary to ensure an accurate identification of hypertension both from the clinical and epidemiological perspective.

## Introduction

Cardiovascular disease (CVD) is the leading cause of mortality worldwide, accounting for over 17 million deaths yearly (1). Among the most prominent risk factors for CVD is early vascular aging, characterized by arterial stiffness (arteriosclerosis) (2). There is extensive evidence that risk factors for CVD occur early in life, causing premature organ damage that tracks into adulthood (3). In fact, arterial stiffness has been observed and related to hypertension in children (4). Hence, early detection of asymptomatic vascular changes such as elevated blood pressure (BP) is essential for effective intervention and prevention of health consequences in older ages.

However, pediatric hypertension has received little attention, especially in low- and middle-income countries (LMICs). As new research emerges, we are gaining awareness of the extent of a long underappreciated problem and the consequences of its (mis)management, such as impaired development, economic burden, years of life lost, etc. (5). A recent meta-analysis revealed that global hypertension rates among children have increased about 75% over the past 20 years (6), which has been partially attributed to the steep escalation in childhood obesity, an early-life risk factor for CVD. A similar trend for childhood hypertension is expected in African countries, where “the number of overweight children under 5 has increased by nearly 24 percent since 2000” (7). The strong association between body mass index (BMI) and BP in children has been established both globally (8) and in South Africa (9), where their relation with arterial stiffness (10) and the tracking of BP from childhood into late adolescence have been described (11). This emphasizes the need to rely on accurate estimates to monitor childhood hypertension progress and react to long-term health impacts, especially in marginalized settings, to inform policy making and ensure meaningful allocation of scarce resources (12). Despite this new knowledge, global attention and prevention efforts are still focused on high-income countries (HICs), while vulnerable populations in LMICs are often neglected.

Several guidelines have established the definition of pediatric hypertension based on normative BP tables that account for age, sex, and height, and settled abnormally elevated BP levels in children at the 90th percentile and hypertension at the 95th percentile (13, 14). Currently, the most widely used guidelines were developed by the American Academy of Pediatrics (AAP) and include normative charts based on normal-weight American children and revised cut-off points (15). However, other efforts to establish BP reference tables have been made. In Germany, reference charts were derived from a population of non-overweight children participating in the KiGGS study, and are broadly used in the literature, as well (16). Xi et al. pooled data from 7 different countries in an attempt to develop universal BP references for children (17). Notwithstanding, no normative tables exist for African children, and these populations have been underrepresented in attempts to develop global references.

Furthermore, it is unclear whether country-specific BP reference tables and uniform international standards are appropriate for the estimation of pediatric hypertension prevalence in different contexts (18). Whereas the percentile values differ between the reference populations used to calculate them, context-specific socioeconomic and environmental factors might make the broad use of such standards unsuitable. Accordingly, studies have shown low consistency between international and local normative tables (19). Indeed, the prevalence of pediatric elevated BP in the African literature varies substantially, and South African studies present conflicting results (8). Different normative data and cut-offs to define high BP are used in these studies, and it remains unclear whether some authors have developed age-, sex-, and height-specific BP reference values based on their own study population, similar to the example from the Gambian study of Modou et al. (20).

Against this background, the current study aims to examine the hypertension prevalence in 897 children from lower-income families in the Eastern Cape province of South Africa considering four different BP references. Subsequently, these BP standards are compared based on their association with increasing BMI-for-age levels, an indicator of poorer cardiovascular health. We hypothesize that international normative tables and charts developed from the same study population will yield different estimates of hypertension prevalence and a different risk profile associated with BMI-for-age among children from marginalized communities in South Africa.

## Materials and Methods

### Study Population

Results are based on cross-sectional analyzes of the *KaziBantu* project cohort baseline assessment, which took place between January and March 2019 (21). Children aged 8–16 years were recruited from eight schools from the economically disadvantaged peri-urban townships and northern areas of Gqeberha, South Africa. All study sites consisted of non-fee paying, quintile 3 schools (South African schools are ranked from quintile one, the poorest, to quintile five, the least poor). In total, 975 children were enrolled in the *KaziBantu* study. Of those, 897 children (449 boys, 448 girls) presented with complete data records, after excluding those participants with missing data for sex (*n* = 8), age (*n* = 9), height (*n* = 47), weight (*n* = 57), and BP (*n* = 31).

### Assessment of Blood Pressure

BP was measured three times on the upper left arm after a seated period of 5 min and with a 1-min rest between readings. A validated, automated oscillometric device (Omron®M6AC; Hoofddorp, Netherlands) and a child appropriate cuff, sized 17–22 cm, were used. Systolic and diastolic BP values were calculated as the mean of the last two readings to avoid overestimation usually observed on the first run.

### Classification of Blood Pressure

Sex-, age-, and height-adjusted normative tables for pediatric BP were used to calculate systolic BP (SBP) and diastolic BP (DBP) percentiles. The following three widely recognized normative data charts were applied by running the programs indicated by each author: (i) AAP 2017 based on an American reference population (15, 22), (ii) Robert Koch-Institute based on a German reference population (16), and (iii) an international reference based on data pooled from 7 countries (China, India, Iran, Korea, Poland, Tunisia, and USA) (17). Furthermore, a fourth reference derived from normal-weight children from the *KaziBantu* study population was used (21). Because the study sample is classified through its own constructed normative tables, the study reference will henceforth be considered pseudo-normative. Details of the reference populations are presented in Supplementary Table S1.

Thereafter, the obtained percentiles were classified as normotension, elevated BP or hypertension stages 1 and 2, for both SBP and DBP, based on the cut-offs suggested in each reference, with the exception of the 99th + 5 mm Hg percentile recommended by Neuhauser et al. for stage 2 hypertension, where we applied a comparable cut-off at the 99.75th percentile to facilitate analysis. The highest value of either SBP or DBP was allocated to determine combined BP.

### Anthropometric Parameters

Body weight was measured on a digital weighing scale; children were barefoot and wore school uniform. Body height was measured against a stadiometer; children were asked to stand still with their back erect and shoulders relaxed. BMI was calculated from both weight and height according to weight (kg)/height (m)^2^. BMI-for-age Z-scores (BAZ) stratified by sex, a measure for obesity, overweight and thinness, were determined according to World Health Organization's (WHO) growth charts (23).

### Statistical Analysis

Descriptive statistics for all variables comprised means (M) and standard deviation (SD). Differences between the means for boys and girls were analyzed via independent *t*-tests for significance and Cohen's d for effect size. Polynomial regressions were used to construct pseudo-normative tables from the *KaziBantu* study population after removing outlying participants and those children whose BMI was higher than 1 SD above the group average. SBP and DBP pseudo-normative charts for boys and girls are available in the Supplementary Table S2. Prevalence of BP categorization is expressed as frequencies (N) and percentages (%) for all four classification standards. Pearson chi-square (χ^2^) tests were carried out to assess the distribution of sex (girls and boys) and age (≤ 10, 10, 11, ≥11 years) in all BP categories for SBP, DBP, and BP combined. Multinomial logistic regression analyzes were used to explore the association of increasing BAZ values with the prediction of BP classification into higher categories.

Statistical tests were performed using IBM SPSS version 26 (IBM; Armonk, New York, USA). Normative BP tables were applied in STATA version 15.1 (StataCorp; College Station, Texas, USA) for the German reference population and in SAS version 9.4 (SAS Institute; Cary, North Carolina, USA) for the American and global references. BP percentile tables of the *KaziBantu* study population were constructed in Statistica version 13 (TIBCO Software Inc., Palo Alto, USA).

## Results

### Descriptive Statistics

Table 1 presents descriptive statistics and characteristics of the study participants, stratified by sex. Girls showed a statistically significant lower mean age (10.2 vs. 10.6 years; *p* < 0.001), while presenting higher mean values for the other variables. Differences in weight (36.7 vs. 34.5 kg), BMI (18.4 vs. 17.5 kg/m^2^), and BAZ (0.2 vs. 0.0) were of statistical significance at the *p* < 0.001 level. However, the Cohen's effect size was small for all sex differences in age (*d* = 0.30), weight (*d* = 0.22), BMI (*d* = 0.25), and BAZ (*d* = 0.21).

**Table 1:** Participant characteristics and comparison between South African girls and boys from the *KaziBantu* study population in Gqeberha, South Africa.

**Parameter**	**Total (*N* = 897)**	**Boys (*N* = 449)**	**Girls (*N* = 448)**		
	**M (SD)**	**M (SD)**	**M (SD)**	***t***	**Cohen's d**
Age (years)	10.4 (1.2)	10.6 (1.2)	10.2 (1.1)	4.48^***^	0.30
SBP^*^ (mm Hg)	108.9 (13.4)	108.6 (13.1)	109.3 (13.7)	−0.86	0.06
DBP^†^ (mm Hg)	67.2 (10.9)	66.5 (11.0)	67.8 (10.8)	−1.74	0.12
Height (cm)	139.9 (8.8)	139.6 (8.7)	140.2 (9.0)	−1.02	0.07
Weight (kg)	35.6 (10.2)	34.5 (9.2)	36.7 (11.0)	−3.29^***^	0.22
BMI^‡^ (kg/m^2^)	18.0 (3.7)	17.5 (3.3)	18.4 (4.0)	−3.74^***^	0.25
BMI-for-age Z-scores	0.1 (1.3)	−0.04 (1.3)	0.2 (1.3)	−3.19^***^	0.21

* *Systolic blood pressure*.

† *Diastolic blood pressure*.

‡ *Body mass index*.

*** *p < 0.001*.

*Cohen's d effect size: d < 0.2: no effect; 0.2 ≤ d < 0.5: small effect; 0.5 ≤ d < 0.8: medium effect; d ≥ 0.8: large effect*.

### Hypertension Prevalence

We classified BP levels according to the four normative charts and their corresponding cut-offs (Table 2). Similar combined hypertension prevalence was observed for the three international references, namely 28.6% based on the American, 28.8% on the German, and 25.6% on the global. In contrast, only 11.4% of children were identified as hypertensive according to the pseudo-normative study reference. The highest level of agreement was found between the American and the global references with 92.5%, whereby 830 children were equally stratified throughout all four categories. The *KaziBantu* classification revealed the highest agreement percentage with the global reference (71.5%) (data not shown).

**Table 2:** Comparison of high blood pressure prevalence among school-aged children in Gqeberha, South Africa, in July 2019 according to the (i) American Academic of Pediatrics, (ii) German guidelines, (iii) a global reference population, and (iv) the *KaziBantu* study population (*N* = 897).

**References**	**Normal blood pressure**	**Elevated blood pressure**	**Hypertension stage 1**	**Hypertension stage 2**
Flynn et al. (15)^*^	555 (61.9%)	85 (9.5%)	181 (20.2%)	76 (8.5%)
Neuhauser et al. (16)^†^	572 (63.8%)	65 (7.2%)	163 (18.2%)	97 (10.8%)
Xi et al. (17)^‡^	565 (63.0%)	102 (11.4%)	159 (17.7%)	71 (7.9%)
Müller et al. (21)^§^	738 (82.3%)	57 (6.4%)	65 (7.2%)	37 (4.1%)

* *Normotension: <13 years old: <90th; >13 years old BP <120/80 mm Hg; elevated BP: <13 years old: ≥90th and <95th or >120/80 mm Hg but <95th; >13 years old: 120/ <80 to 129/ <80 mm Hg; HTN stage 1: <13 years old: ≥95th and <95th + 12 mm Hg or 130/80–139/89 mm Hg; >13 years old: 130/80 mm Hg to 139/89 mm Hg; HTN stage 2: <13 years old: ≥95th + 12 mm Hg or ≥140/90 mm Hg; >13 years old: ≥140/90 mm Hg*.

† *Normotension: <90th; elevated BP: ≥90th and <95th; HTN stage 1: ≥95th and <99.75th; HTN stage 2: ≥99.75th or ≥140/90 mmHg*.

‡ *Normotension: <90th; elevated BP: ≥90th and <95th or >120/80 mm Hg but <95th; HTN stage 1: ≥95th and <99th + 5 mm Hg; HTN stage 2: ≥99th + 5 mm Hg*.

§ *Normotension: <90th; elevated BP: ≥90th and <95th; HTN stage 1: ≥95th and <95th+12 mm Hg; HTN stage 2: ≥95th + 12 mm Hg*.

Sub-analyzes showed that the biggest disagreement between the study reference and international guidelines lied in the classification of SBP. While hypertension was more than three times higher for SBP with the American classification compared to *KaziBantu*'s (24.2 vs. 7.2%), it was almost double for DBP (14.7 vs. 7.7%). Thus, the main driver for hypertension when applying the American guidelines was SBP rather than DBP. The higher systolic over diastolic hypertension prevalence was also observed with the global reference (23.6% for SBP vs. 10.6% for DBP).

All four standards showed similar distributions between girls and boys regardless of systolic, diastolic, or combined BP. Only in the American reference, a significant association between sex and DBP classification was observed [$\chi_{\left(3\right)}^{2}$ = 10.46, *p* = 0.015], whereby girls were disproportionately associated with hypertension stage 1 (64.6%) at the *p* = 0.05 significance level. Age showed no association with SBP, DBP, or combined BP categorization for any of the references. Sub-analyzes can be found in the Supplementary Tables S3–S5.

### Reference Populations Comparison Based on BMI-For-Age

**Figure 1:**
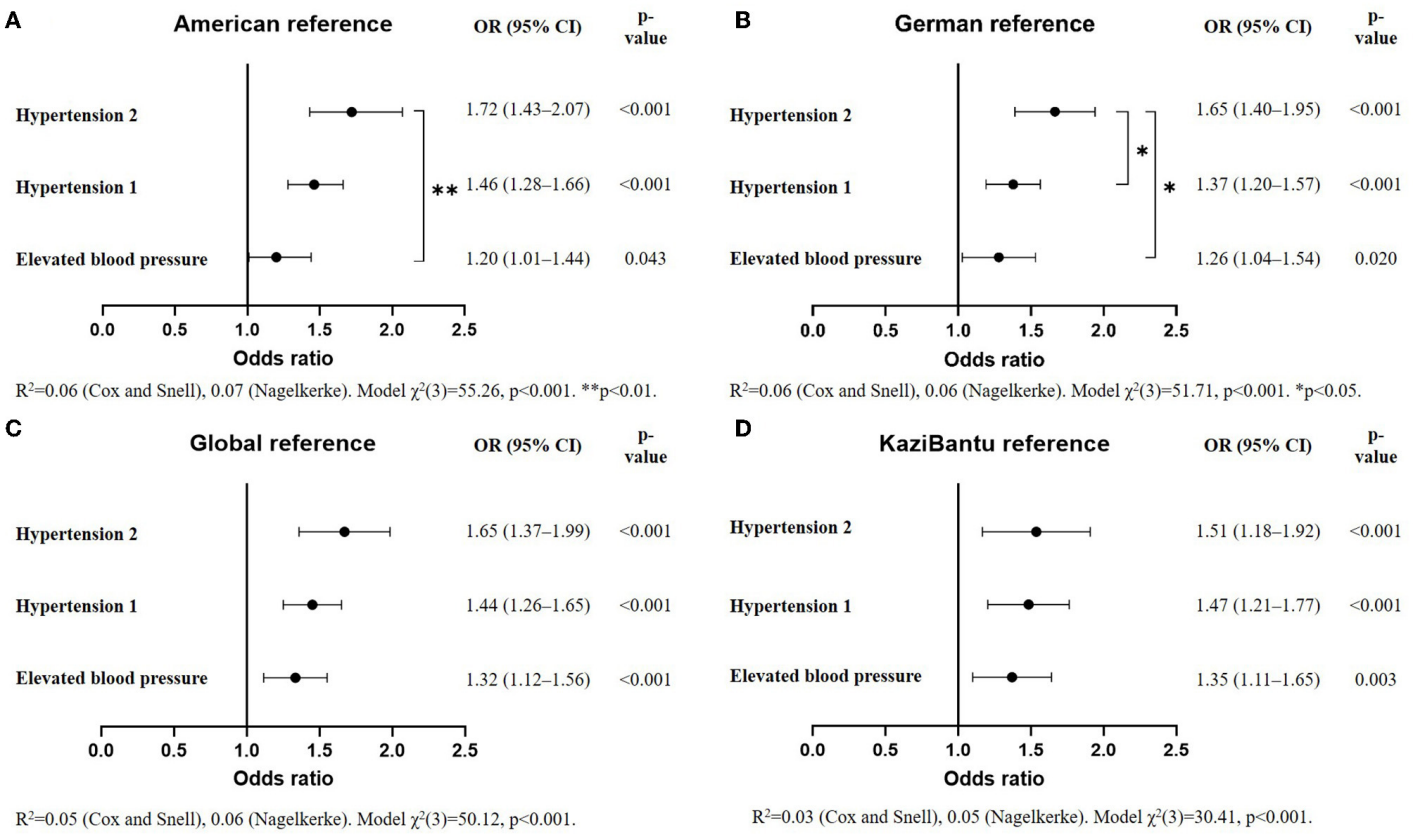
Odds ratio (OR) of the classification of blood pressure (BP) as elevated, hypertension stage 1 or stage 2, relative to normotension, with increasing BMI-for-age Z-scores according to **(A)** American, **(B)** German, **(C)** global, and **(D)** the *KaziBantu* reference populations (*N* = 897).

The association between being classified as hypertensive with higher BMI levels for all studied references is illustrated in Figure 1. All four standards showed a significant increased risk for being categorized as having elevated BP, hypertension stage 1, and hypertension stage 2 per standard deviation increase in BMI-for-age relative to the normotensive group. The highest odds for hypertension stage 2 with increasing BAZ was seen with the American guidelines with a 72% increased risk (95% CI: 1.43–2.07; *p* < 0.001), followed by a 65% increased risk with both the global (95% CI: 1.37–1.99; *p* < 0.001) and German references (95% CI: 1.40–1.95; *p* < 0.001). The lowest odds were found with the pseudo-normative *KaziBantu* reference at 51% (95% CI: 1.18–1.92; *p* < 0.001). The increased risk for hypertension stage 1 with increasing BAZ was similar across the American, global, and *KaziBantu* study references at ~45%, being lowest with the German classification (OR = 1.37; 95% CI: 1.20–1.57; *p* < 0.001). Further sub-analyzes are shown in Supplementary Table S6. A significant increased risk of 43% (*p* = 0.003) per unit increase in BAZ was found between elevated BP and hypertension stage 2 for the American reference. With the German guidelines, increased categorization risk was significant between elevated BP and hypertension stage 2 (OR = 1.31, *p* = 0.027), and between hypertension stage 1 and hypertension stage 2 (OR = 1.20, *p* = 0.05). No significant differences were observed for the global and *KaziBantu* references.

## Discussion

This study demonstrates differences in the implementation of international standards for the classification of BP in diverse contexts. Among school-aged children from disadvantaged communities in the Eastern Cape Province of South Africa, the obtained prevalence of hypertension ranged from 11.4%, when pseudo-normative tables from the own study population were used, to 28.8% based on international standards. Further analyzes show that the risk of being categorized as hypertensive with increasing levels of BAZ, a marker for adiposity, also varied among these standards, with the highest risk observed when international references were applied.

The first aim of the study was to establish and compare the prevalence of elevated BP and hypertension based on HIC standards, a pooled global reference, and pseudo-normative tables derived from the *KaziBantu* study population. We encountered a pronounced variability when different normative data were employed. International guidelines yielded the highest prevalence of hypertension in our study population at almost 30%. Although South Africa presents a higher hypertension prevalence than other sub-Saharan African countries, attributed partly to differences in countries' socioeconomic development (24), the estimates in the present study lie far above those reported by other authors. In fact, only one study has shown a higher combined hypertension prevalence of 32.6% (25). Interestingly, the authors used the German reference population from Neuhauser et al. (26). In contrast, when the *KaziBantu* pseudo-normative reference was used, hypertension prevalence was almost three times lower (11.4%) and thus, closer to the estimations found in the literature. However, they still remain higher than the 7.9% prevalence rates reported for Southern Africa (27) and the 8.1% for South Africa (8) in similar age groups. Nevertheless, it is noteworthy that a considerable heterogeneity exists within the South African literature, where pediatric hypertension ranges from 2.6 to 20.7% (28, 29).

This variability might be partly explained by methodological factors, such as the use of different instruments (electronic devices, sphygmomanometer, and finger-arterial pressure apparatus), or the number and occasions of BP readings (lowest of three readings, average of last two of three readings, average of three out of five measurements with the smallest variation). Arguably, we believe that this disparity is particularly governed by the use of different categorization standards and cut-offs. Many South African authors have based their estimations on outdated guidelines (29, 30), potentially leading to an underestimation of prevalence rates (31). Others have made use of the updated AAP 2017 guideline by applying simplified cut-offs. For example, Matjuda et al. considered exclusively percentiles disregarding whole values (in mm Hg) and age differences (<13 vs. >13 years) (10). In some studies, it is unclear whether researchers have developed pseudo-normative percentiles based on their own study population and subsequently classified them according to international standards (11, 32). Other studies do not describe the analysis altogether (33). Our results are in line with the disparities in hypertension estimates found in the literature and highlight an unstandardized use of methods and classification guidelines.

The discussion around the accuracy of hypertension estimates should however focus on their clinical significance, that is, on establishing BP levels that relate to a real risk for early organ damage and CVD. Hence, other environmental and socioeconomic factors that influence BP should also be taken into account when applying standards that aim at stratifying risk across different contexts. Contrary to common belief, two recent meta-analyzes have shown that hypertension was more prevalent among LMICs than HICs (34, 35). The most affected are middle-transitioning economies like South Africa, which through unplanned and rapid urbanization are adopting westernized lifestyles, while communicable diseases continue to thrive (36). In fact, infections are a cause of undernutrition, anemia, and growth retardation, which, in turn, have been associated with hypertension (37). Similarly, lower socioeconomic status has been associated with higher prevalence of CVD risk factors (38) and hypertension (39). Kagura et al. corroborated these findings in adolescents from the township of Soweto in South Africa, showing a protective role for SBP with transition from low to high socioeconomic status (40). The previously exposed suggests that particular sociological, economic, and demographic characteristics account for the variability in the pattern of CVD risk factors and justify questioning the accuracy and significance of hypertension estimates, as well as their generalizability.

The second study aim was to evaluate whether the association of BMI with BP categorization differed between international references and pseudo-normative percentiles derived from the studied population, for the relation between obesity and elevated BP in African children has been extensively described in the literature (41–43). In the “Birth to Twenty” cohort from Soweto, adiposity in early life associated with the later development of elevated BP in black adolescents (44). Kruger et al. have reported a 60% increased risk of having hypertension per unit increase in BAZ in children from the Western Cape, South Africa (9). In our sample, American guidelines showed the highest increased risk for hypertension stage 2 at 72% compared to 51% with the *KaziBantu* pseudo-normative reference. In our view, the association of increasing BAZ levels with a higher risk for being ranked into upper BP categories obtained with the American guidelines speaks for a potentially better classification.

Furthermore, ~50% of hypertension participants in the American and global references were hypertensive due to isolated systolic hypertension (ISH) compared to isolated diastolic and combined hypertension. This difference was not observed with the *KaziBantu* pseudo-normative reference. In the last years, SBP has gained relevance as a standalone risk factor for cardiovascular morbidity and mortality in adults (45). The primary prognostic significance of ISH has been observed in children too (46). Concluding, in our sample CVD risk understood as increased BMI-for-age levels and ISH was better predicted by the American and global guidelines.

### Limitations

The results of the present study must be considered in light of the following limitations. First, international guidelines suggest that clinical hypertension needs to be confirmed as a high BP on three separate occasions since the prevalence of elevated BP tends to decrease over subsequent visits. In line with other epidemiological studies, we have measured BP on one visit and discarded the first reading to avoid an overestimation of results. Still, Kruger et al. stated that “by using three BP measurements during a single visit and averaging either all or the last two measurements, provides larger statistical variance and yields higher BP averages” (p. 8) (9).

Second, the study population has a specific profile of children living in low-income, urban areas of the Eastern Cape of South Africa, and is thus, not representative of the broad demographic spectrum characteristic of the population of South Africa. Furthermore, we had a relatively small population size, while simplified normative BP tables were calculated due to an unequal distribution of age. It is worth mentioning that while American and global guidelines both consist of a sample size of around 50,000 children, the relative small sample size of the *KaziBantu* study reference may have led to an underestimation of the hypertension prevalence and a weaker risk prediction potential.

Third, this study evaluated solely the association between BP and BMI to assess risk prediction of the standards, but did not collect data on other determinants of health that influence BP. Moreover, the design of the study does not allow to link BP levels to future adverse health events. Thus, it is not possible to draw definitive conclusions about suitability, or superiority, of any of the standards.

Our findings underscore the need for further research into the medical significance of current BP management guidelines in Southern Africa. Prospective longitudinal studies will be required to associate BP levels in childhood with the incidence of CVD and mortality later in life, and to establish clinically relevant cut-off values.

## Conclusion

We found differences in hypertension prevalence depending on the normative BP tables applied to a population of 8- to 16-year-old children from disadvantaged communities in South Africa. Furthermore, we portray a marked underrepresentation of African children in international guidelines and a need for the standardization of BP data collection and analysis in this region. Hence, we advocate for the development of normative tables that are representative of the (South) African pediatric population (and other distinct populations currently not represented in panels informing international guidelines) and the definition of cutoffs that are based on clinical evidence to ensure accurate identification of hypertension both from the clinical and epidemiological perspective in marginalized populations. We conclude that until national or regional guidelines exist, large-enough international reference populations may be used in contexts different from the ones they were developed, albeit findings must be interpreted with caution.

## Data Availability Statement

The raw data supporting the conclusions of this article will be made available by the authors, without undue reservation.

## Ethics Statement

The studies involving human participants were reviewed and approved by Nelson Mandela University Ethics Committee (H18-HEA-HMS-001), Eastern Cape Department of Education, Eastern Cape Department of Health, Ethics Committee (EC_201804_00), and Northwest and Central Switzerland (R-2018-00047). Written informed consent to participate in this study was provided by the participants' legal guardian/next of kin, while oral assent was sought from children.

## Author Contributions

PA research idea, statistical analysis and interpretation of data, and draft preparation. JB and HS conceptualization and statistical analysis. IM, MG, and HS preparation of manuscript. LA, JD, DD, SG, NJ, IM, MN, and SN data collection and curation. All authors critical revision of the manuscript, provided approval for publication of the content, and agree to be accountable for the content of the work.

## Funding

The work presented here was financially supported by the Novartis Foundation (Basel, Switzerland) and the Swiss National Science Foundation (Bern, Switzerland; grant no. 192651), and took place under the auspices of the UNESCO Chair on Physical Activity and Health in Educational Settings. The funders had no role in study design, data collection, data analysis or data interpretation, nor the decision to submit the manuscript for publication.

## Conflict of Interest

AA was employed by the company Novartis Foundation. The remaining authors declare that the research was conducted in the absence of any commercial or financial relationships that could be construed as a potential conflict of interest.

## Publisher's Note

All claims expressed in this article are solely those of the authors and do not necessarily represent those of their affiliated organizations, or those of the publisher, the editors and the reviewers. Any product that may be evaluated in this article, or claim that may be made by its manufacturer, is not guaranteed or endorsed by the publisher.
