# Supplementary material for: Practice Change Needed for the Identification of Pediatric Hypertension in Marginalized Populations: An Example From South Africa
Source: Front Pediatr. 2022 May 11;10:877431. doi: 10.3389/fped.2022.877431 (PMC9130957; doi:10.3389/fped.2022.877431)
Supplement: Supplementary file 1 [file Table_1.DOCX]

Supplementary Material

# Supplementary Table S1

| **Table S1 \|** Characteristics of the reference populations including number of children, age range, ethnicity, time period, instrument used and number of readings | | | | | | |
| --- | --- | --- | --- | --- | --- | --- |
| Reference population | N | Age | Ethnicity | Time period | Instrument | Number of readings |
| United States | 49,967 | 1-17 | Mixed; African-American 29% | 1973 - 2000 | Mercury sphyngomanometer | Varied; analysis based on first reading |
| Germany | 12,199 | 3 - 17 | White | 2003 - 2006 | Automated oscillometric device | Mean of two readings |
| Global | 52,636 | 6 - 19 | Mixed, African-American approx. 5% | 1997 - 2013 | Mercury sphyngomanometer | Varied; analysis based on mean of last two readings |
| *KaziBantu* | 897 | 8 - 16 | African; black and coloured | 2019 | Automated oscillometric device | Mean of last 2 readings |

# Supplementary Table S2

## Supplementary Table S2.A

| **Table S2.A \|** Normative tables for systolic blood pressure (SBP) according to age and height for girls from the *KaziBantu* study | | | | | | |
| --- | --- | --- | --- | --- | --- | --- |
| **Age** | **SBP** | **Height** | | | | |
|  |  | 10th | 25th | 50th | 75th | 90th |
|  |  | 124.4 | 129.0 | 133.6 | 137.6 | 140.8 |
| 9 | 50th | 104 | 104 | 104 | 105 | 106 |
|  | 90th | 121 | 121 | 121 | 122 | 123 |
|  | 95th | 126 | 126 | 126 | 127 | 128 |
|  | 95th +12 | 138 | 138 | 138 | 139 | 140 |
|  |  | 10th | 25th | 50th | 75th | 90th |
|  |  | 131.5 | 134.5 | 140.6 | 144.7 | 150.0 |
| 10 | 50th | 104 | 104 | 106 | 108 | 111 |
|  | 90th | 121 | 121 | 123 | 125 | 128 |
|  | 95th | 126 | 126 | 128 | 130 | 133 |
|  | 95th +12 | 138 | 138 | 140 | 142 | 145 |
|  |  | 10th | 25th | 50th | 75th | 90th |
|  |  | 135.0 | 140.1 | 145.3 | 149.6 | 153.0 |
| 11 | 50th | 105 | 106 | 106 | 111 | 113 |
|  | 90th | 122 | 123 | 123 | 128 | 130 |
|  | 95th | 127 | 128 | 128 | 133 | 135 |
|  | 95th +12 | 139 | 140 | 140 | 145 | 147 |
|  |  | 10th | 25th | 50th | 75th | 90th |
|  |  | 137.5 | 143.3 | 148.4 | 152.9 | 157.0 |
| 12 | 50th | 105 | 108 | 110 | 114 | 117 |
|  | 90th | 122 | 125 | 127 | 131 | 134 |
|  | 95th | 127 | 130 | 132 | 136 | 139 |
|  | 95th +12 | 139 | 142 | 144 | 148 | 151 |

## Supplementary Table S2.B

| **Table S2.B \|** Normative tables for diastolic blood pressure (DBP) according to age and height for girls from the *KaziBantu* study | | | | | | |
| --- | --- | --- | --- | --- | --- | --- |
| **Age** | **DBP** | **Height** | | | | |
|  |  | 10th | 25th | 50th | 75th | 90th |
|  |  | 124.4 | 129.0 | 133.6 | 137.6 | 140.8 |
| 9 | 50th | 65 | 64 | 64 | 64 | 64 |
|  | 90th | 78 | 77 | 77 | 77 | 77 |
|  | 95th | 82 | 81 | 81 | 81 | 81 |
|  | 95th +12 | 94 | 93 | 93 | 93 | 93 |
|  |  | 10th | 25th | 50th | 75th | 90th |
|  |  | 131.5 | 134.5 | 140.6 | 144.7 | 150.0 |
| 10 | 50th | 64 | 64 | 65 | 65 | 67 |
|  | 90th | 77 | 77 | 78 | 78 | 80 |
|  | 95th | 81 | 81 | 81 | 82 | 84 |
|  | 95th +12 | 93 | 93 | 93 | 94 | 96 |
|  |  | 10th | 25th | 50th | 75th | 90th |
|  |  | 135.0 | 140.1 | 145.3 | 149.6 | 153.0 |
| 11 | 50th | 65 | 65 | 66 | 67 | 69 |
|  | 90th | 77 | 78 | 79 | 80 | 82 |
|  | 95th | 81 | 82 | 83 | 84 | 85 |
|  | 95th +12 | 93 | 94 | 95 | 96 | 97 |
|  |  | 10th | 25th | 50th | 75th | 90th |
|  |  | 137.5 | 143.3 | 148.4 | 152.9 | 157.0 |
| 12 | 50th | 105 | 108 | 110 | 114 | 117 |
|  | 90th | 122 | 125 | 127 | 131 | 134 |
|  | 95th | 127 | 130 | 132 | 136 | 139 |
|  | 95th +12 | 139 | 142 | 144 | 148 | 151 |

## Supplementary Table S2.C

| **Table S2.C \|** Normative tables for systolic blood pressure (SBP) according to age and height for boys from the *KaziBantu* study | | | | | | |
| --- | --- | --- | --- | --- | --- | --- |
| **Age** | **SBP** | **Height** | | | | |
|  |  | 10th | 25th | 50th | 75th | 90th |
|  |  | 124.8 | 127.5 | 131.6 | 135.0 | 139.2 |
| 9 | 50th | 107 | 106 | 105 | 105 | 105 |
|  | 90th | 123 | 122 | 121 | 121 | 121 |
|  | 95th | 128 | 127 | 126 | 126 | 126 |
|  | 95th +12 | 140 | 139 | 138 | 138 | 138 |
|  |  | 10th | 25th | 50th | 75th | 90th |
|  |  | 129.3 | 133.5 | 136.9 | 141.2 | 143.6 |
| 10 | 50th | 106 | 105 | 105 | 105 | 106 |
|  | 90th | 122 | 121 | 121 | 121 | 122 |
|  | 95th | 127 | 126 | 126 | 126 | 127 |
|  | 95th +12 | 139 | 138 | 138 | 138 | 139 |
|  |  | 10th | 25th | 50th | 75th | 90th |
|  |  | 132.7 | 136.3 | 140.4 | 145.7 | 151.0 |
| 11 | 50th | 105 | 105 | 105 | 107 | 109 |
|  | 90th | 121 | 121 | 121 | 123 | 126 |
|  | 95th | 126 | 126 | 126 | 128 | 130 |
|  | 95th +12 | 138 | 138 | 138 | 140 | 142 |
|  |  | 10th | 25th | 50th | 75th | 90th |
|  |  | 138.0 | 141.0 | 146.2 | 152.3 | 155.4 |
| 12 | 50th | 105 | 106 | 107 | 110 | 113 |
|  | 90th | 121 | 122 | 123 | 126 | 129 |
|  | 95th | 126 | 126 | 128 | 131 | 133 |
|  | 95th +12 | 138 | 138 | 140 | 143 | 145 |

## Supplementary Table S2.D

| **Table S2.D \|** Normative tables for diastolic blood pressure (DBP) according to age and height for boys from the *KaziBantu* study | | | | | | |
| --- | --- | --- | --- | --- | --- | --- |
| **Age** | **SBP** | **Height** | | | | |
|  |  | 10th | 25th | 50th | 75th | 90th |
|  |  | 124.8 | 127.5 | 131.6 | 135.0 | 139.2 |
| 9 | 50th | 65 | 64 | 64 | 63 | 63 |
|  | 90th | 78 | 78 | 77 | 76 | 76 |
|  | 95th | 82 | 81 | 81 | 80 | 80 |
|  | 95th +12 | 94 | 93 | 93 | 92 | 92 |
|  |  | 10th | 25th | 50th | 75th | 90th |
|  |  | 129.3 | 133.5 | 136.9 | 141.2 | 143.6 |
| 10 | 50th | 64 | 63 | 63 | 63 | 63 |
|  | 90th | 77 | 76 | 76 | 76 | 76 |
|  | 95th | 81 | 80 | 80 | 80 | 80 |
|  | 95th +12 | 93 | 92 | 92 | 92 | 92 |
|  |  | 10th | 25th | 50th | 75th | 90th |
|  |  | 132.7 | 136.3 | 140.4 | 145.7 | 151.0 |
| 11 | 50th | 63 | 63 | 63 | 63 | 64 |
|  | 90th | 77 | 76 | 76 | 76 | 77 |
|  | 95th | 80 | 80 | 80 | 80 | 81 |
|  | 95th +12 | 92 | 92 | 92 | 92 | 93 |
|  |  | 10th | 25th | 50th | 75th | 90th |
|  |  | 138.0 | 141.0 | 146.2 | 152.3 | 155.4 |
| 12 | 50th | 63 | 63 | 63 | 64 | 65 |
|  | 90th | 76 | 76 | 76 | 77 | 78 |
|  | 95th | 80 | 80 | 80 | 81 | 82 |
|  | 95th +12 | 92 | 92 | 92 | 93 | 94 |

# Supplementary Table S3

| **Table S3** **\|** Comparison of high systolic (SBP) and diastolic blood pressure (DBP) prevalence among school-aged children in Gqeberha, South Africa, in July 2019 according to (i) the American Academic of Paediatrics, (ii) German guidelines, (iii) a global reference population and (iv) the *KaziBantu* study population (N=897) | | | | | | | | |
| --- | --- | --- | --- | --- | --- | --- | --- | --- |
| **Guideline** | **Normal blood pressure** | | **Elevated blood pressure** | | **Hypertension**  **stage 1** | | **Hypertension**  **stage 2** | |
|  | **SBP** | **DBP** | **SBP** | **DBP** | **SBP** | **DBP** | **SBP** | **DBP** |
| Flynn et al. (15)^*^ | 594 (66.2%) | 721 (80.4%) | 86  (9.6%) | 44  (4.9%) | 154 (17.2%) | 96 (10.7%) | 63  (7.0%) | 36  (4.0%) |
| Neuhauser et al. (16)^†^ | 652 (72.7%) | 689 (76.8%) | 51  (5.7%) | 49  (5.5%) | 132 (14.7%) | 95 (10.6%) | 62  (6.9%) | 64  (7.1%) |
| Xi et al. (17)^‡^ | 598 (66.7%) | 741 (82.6%) | 87  (9.7%) | 61  (6.8%) | 149 (16.6%) | 62  (6.9%) | 63  (7.0%) | 33  (3.7%) |
| *KaziBantu* (21)^§^ | 784 (87.4%) | 794 (88.5%) | 48  (5.4%) | 34  (3.8%) | 45  (5.0%) | 41  (4.6%) | 20  (2.2%) | 28  (3.1%) |

*Normotension: <13 years old: <90th; >13 years old BP<120/80 mmHg; elevated BP: <13 years old: ≥90th and <95th or >120/80 mmHg but <95th; >13 years old: 120/<80 to 129/<80 mmHg; HTN stage 1: <13 years old: ≥95th and <95th+12mmHg or 130/80 mmHg to 139/89 mmHg; >13 years old: 130/80 mmHg to 139/89 mmHg; HTN stage 2: <13 years old: ≥95th+12mmHg or ≥140/90 mmHg; >13 years old: ≥140/90 mmHg.

†Normotension: <90th; elevated BP: ≥90th and <95th; HTN stage 1: ≥95th and <99.75th; HTN stage 2: ≥99.75th or ≥140/90 mmHg.

‡Normotension: <90th; elevated BP: ≥90th and <95th or >120/80 mmHg but <95th; HTN stage 1: ≥95th and <99th+5mmHg; HTN stage 2: ≥99th+5mmHg.

§Normotension: <90th; elevated BP: ≥90th and <95th; HTN stage 1: ≥95th and <95th+12mmHg; HTN stage 2: ≥95th+12mmHg.

# Supplementary Table S4

| **Table S4** **\|** Pearson chi-square test for association of sex with categorization of systolic (SBP), diastolic (DBP) and combined blood pressure according to the (i) American, (ii) German guidelines, (iii) a global reference population and (iv) the *KaziBantu* study population | | | | | | |
| --- | --- | --- | --- | --- | --- | --- |
| **Guideline** | **SBP** | | **DBP** | | **Combined** | |
|  | ꭕ^2*^ | p | ꭕ^2*^ | p | ꭕ^2*^ | p |
| Flynn et al. (15) | 9.38 | 0.403 | 13.68 | 0.134 | 6.55 | 0.684 |
| Neuhauser et al. (16) | 5.84 | 0.756 | 8.48 | 0.487 | 4.64 | 0.865 |
| Xi et al. (17) | 2.28 | 0.986 | 6.73 | 0.665 | 3.29 | 0.952 |
| *KaziBantu* (21) | 9.96 | 0.354 | 9.65 | 0.379 | 11.77 | 0.227 |

*(df=3)

# Supplementary Table S5

| **Table S5** **\|** Pearson chi-square test for association of age with categorization of systolic (SPB), diastolic (DBP) and combined blood pressure according to the (i) American, (ii) German guidelines, (iii) a global reference population and (iv) the *KaziBantu* study population | | | | | | |
| --- | --- | --- | --- | --- | --- | --- |
| **Guideline** | **SBP** | | **DBP** | | **Combined** | |
|  | ꭕ^2*^ | p | ꭕ^2*^ | p | ꭕ^2*^ | p |
| Flynn et al. (15) | 9.38 | 0.403 | 10.46 | 0.015 | 1.72 | 0.633 |
| Neuhauser et al. (16) | 2.16 | 0.540 | 5.20 | 0.158 | 1.22 | 0.747 |
| Xi et al. (17) | 1.29 | 0.731 | 2.31 | 0.511 | 0.74 | 0.864 |
| *KaziBantu* (21) | 2.19 | 0.535 | 2.28 | 0.516 | 0.62 | 0.892 |

*(df=3)

# Supplementary Table S6

## Supplementary Table S6.A

| **Table S6.A** **\|** Odds ratio (OR) of the classification of blood pressure as normal, hypertension stage 1 or stage 2 relative to elevated blood pressure with increasing BMI-for-age Z-scores according to the American reference (N=897) | | |
| --- | --- | --- |
| **Category** | **OR (95% CI)** | **p-value** |
| Normal blood pressure | 0.83 (0.69 – 0.99) | 0.043 |
| Hypertension stage 1 | 1.21 (0.99 – 1.48) | 0.059 |
| Hypertension stage 2 | 1.43 (1.13–1.81) | 0.003 |

## Supplementary Table S6.B

| **Table S6.B** **\|** Odds ratio (OR) of the classification of blood pressure as normal, elevated or hypertension stage 2 relative to hypertension stage 1 with increasing BMI-for-age Z-scores according to the American reference (N=897) | | |
| --- | --- | --- |
| **Category** | **OR (95% CI)** | **p-value** |
| Normal blood pressure | 0.67 (0.60 – 0.78) | <0.001 |
| Elevated blood pressure | 0.83 (0.68 – 1.01) | 0.059 |
| Hypertension stage 2 | 1.18 (0.97–1.44) | 0.099 |

## Supplementary Table S6.C

| **Table S6.C** **\|** Odds ratio (OR) of the classification of blood pressure as normal, hypertension stage 1 or stage 2 relative to elevated blood pressure with increasing BMI-for-age Z-scores according to the German reference (N=897) | | |
| --- | --- | --- |
| **Category** | **OR (95% CI)** | **p-value** |
| Normal blood pressure | 0.79 (0.65–0.96) | 0.021 |
| Hypertension stage 1 | 1.08 (0.88–1.35) | 0.468 |
| Hypertension stage 2 | 1.31 (1.03–1.66) | 0.027 |

## Supplementary Table S6.D

| **Table S6.D** **\|** Odds ratio (OR) of the classification of blood pressure as normal, elevated or hypertension stage 2 relative to hypertension stage 1 with increasing BMI-for-age Z-scores according to the German reference (N=897) | | |
| --- | --- | --- |
| **Category** | **OR (95% CI)** | **p-value** |
| Normal blood pressure | 0.73 (0.64 – 0.84) | <0.001 |
| Elevated blood pressure | 0.92 (0.74 – 1.15) | 0.468 |
| Hypertension stage 2 | 1.20 (1.00 – 1.45) | 0.050 |
